# Practical Notes and Formulæ

**Published:** 1880-08-20

**Authors:** 


### PRACTICAL NOTES AND FORMULÆ.

**The Vomiting of Pregnancy.**—A medical friend of Iowa writes:

In the July number of the Record I see a remedy for nausea in pregnancy, as well as useful in flatulent dyspepsia, for which it was first suggested by Dr. Wood. U.S.D., and methinks Dr. Wood knew about all that it was good for, and gave us the benefit of that knowledge when he recommends it for the latter trouble.

He says we are in the habit of prescribing two parcels of the mixture to take continuously, mixing one at a time. This quantity is sufficient in mild cases, but of course it must be continued longer if necessary, and if the complaint returns in the course of three or four weeks a few pints more will be needed, or, in other words, you give the pregnant woman a placebo and let her worry along until her enlarged womb rises from its cramped position in the pelvis, the irritated nerves are relieved and the nausea and vomiting subside.

I suggest that the Dr.—the next case he is called to treat of the kind—use bromide potass. per enema, and a little ingluvin for the stomach, as suggested in the Record a year or more ago, and he will give the roots to the dyspepsia, as Dr. Wood directs, hereafter.

**Hay Fever.**—Dr. R. H. Weber, of Philadelphia, communicates to the American Journal of Pharmacy a copy of the prescription which he has uniformly found useful in the complaint mentioned. Dr. Weber regards the iodide of potassium as the active agent, but the best results have always been obtained when combined with bicarbonate of potassium and hyoscyamus. The formula is as follows:

℞ Extracti hyoscyami............................................ gr. xij<br>

Potassii iodidi........................................... ʒj<br>

Potassii bicarbonatis........................................... ʒij<br>

Ext. glycyrrhizæ depurati........................................... ʒiv<br>

Aquæ anisi........................................... ℥ivss M

Dose, a dessertspoonful every four hours, day and night, until relieved. The medicine is to be continued for at least a week, in doses of a dessertspoonful four times daily.—*Southern Practitioner.*

**For External Piles.**—

℞ Unguenti gallæ comp........................<br>

Extracti belladonnæ........................aa equal parts. M

Dr. David Young, of Florence, says, in the Practitioner, that of all the plans which he has adopted, none have yielded such satisfactory results as the following, viz.:

To bathe the part thoroughly with water, as warm as can be borne, together with the free use of Castile soap, and afterward to apply the above ointment. The operation must be repeated every three or four hours, till the pain subsides. Usually the first application gives great relief. Without the previous washing with soap and warm water the application of the ointment is of little service.—*Med. and Surg. Rep.*

**Alcohol and Creosote in Infants' Diarrhœa.**—We translate from the *Journal de Medecine et de Chirurgie Pratiques* :

In the diarrhœa of infants submitted to a too early nutrition, Dr. Demme, of Berne, not only recommends the breast at the exclusion of other aliments, but he also recommends alcohol, combined either with benzoate of soda or with creosote.

| | | |
|---|---|---|
| ℞ | Cognac................................ | 2 to 5 grammes. |
| | Creosote ................................ | 0,01 centigramme. |
| | Tar...................................... | 1 to 5 grammes. |
| | Aq. dist................................ | 50 grammes. |

To be taken every twenty-four hours between sucking.

In very young infants he begins with two grammes of alcohol which he gradually increases to five grammes.

The effect of this prescription is to stimulate nutrition and prevent the formation of too many micrococcus in the intestinal glandulas.

**To Relieve Itching.**—

| | | | |
|---|---|---|---|
| ℞ | Acidi carbolici.............................. | gtt. x | |
| | Glycerinæ.................................... | ʒj | |
| | Aquam, ad.................................... | ℥j | M |

Sig. To be used in an atomizer, five minutes at a time, several times daily.

This is used by Dr. Rigault, of Paris, in itching from all causes, in lichen, eczema, prurigo, etc., and with very general success wherever the irritation is nervous rather than mechanical.—*Med. and Surg. Rep.*

**Tonic Glycerine.**—Where cod-liver oil cannot be tolerated, the following "tonic glycerine" may be substituted :

| | | |
|---|---|---|
| ℞ | Pure glycerine.................................. | ℥x |
| | Tinct. iodine.................................... | gtts. xxx |
| | Potass. iodid .................................... | gr. v |

Dose, a tablespoonful a quarter of an hour before each meal.—*Med. and Surg. Reporter.*

**Application for painful Hemorrhoids.**—The Magazine of Pharmacy gives the following as a valuable application for painful hemorrhoids :

| | | |
|---|---|---|
| ℞ | Extract of henbane.............................. | ʒ 2½ |
| | Powdered saffron................................ | ʒ 2½ |
| | Acetate of lead.................................. | ʒ 1 |
| | Glycerole of starch.............................. | ℥ 1 |

Mix and apply to the parts three or four times a day.

**Fetid Perspiration of the Feet.**—Dr. Ortega, in *La Gazette Medicale de l'Algerie*, reports a case of a man with exceedingly offensive perspiration of the feet. A simple washing in a solution of chloral, one per cent., followed by the wrapping up of the feet in a cloth saturated with the same solution immediately and completely removed the fetidity.

**Cholera and Diarrhœa.**—Dr. Permar, in Lancet and Clinic, recommends the following:

℞ Tannic acid........ ʒj

Creta prep........ ʒj

Jamaica ginger........ ʒiij

Tinct. peppermint........ ℥j

Tinct. opii........ ʒij

Tinct. camphor........ ℥j

Brandy, best........ ℥vi

Dose, a teaspoonful every three hours. A good remedy in bowel troubles.—*Ex.*

**For Amenorrhœa from Torpidity of the Ovaries.**—Prof. Goodell recommends the following:

℞ Ext. aloes........ ʒj

Ferri sulph, exsic........ ʒij

Assafet........ ʒiv

Mix and make into 100 pills. Dose, one pill after each meal, to be gradually increased to two, and then to three pills after each meal. Lessen the dose when the bowels begin to act too freely.—*Ex.*

**Enema for Dysentery.**—When ipecacuanha cannot be given by the mouth, Surgeon W. King says, in the Lancet, he has derived the greatest advantage from the following:

℞ Bismuthi subnitratis........ ℈ij

Tinct. opii........ ʒss

Pulv. ipecac........ ℈ij

Mucilaginis........ ℥iij M

Sig. For an enema, to be thrown up after first gently cleansing the bowels by an enema of lukewarm water.—*Med. and Surg. Reporter.*

**Chronic Constipation.**—Dr. Clark, in a paper read before Wayne County Medical Society, suggests the following formula as excellent in obstinate chronic constipation:

℞ Ext. Rhamni, Purch. (Cascara)........ ℥j

Ext. Belladonna........ ʒj

Tinct. nux vomica........ ʒij

Syrupi et aquæ, aa ad........ ℥iv

Dose, a teaspoonful thrice daily.

**Remedies for Purpura.**—Five drops of muriatic acid in a wine-glass of water three times a day, alternated with teaspoonful doses of muriated tincture of iron, has been highly recommended.

℞ Glycerine........ ℥iv

Tinct. digitalis........ ʒiij

Dilute phosphoric acid........ ʒiij M

Dose, a teaspoonful three times a day.

This is often very prompt in controling the hemorrhage, but its administration requires caution.—*N. Y. Med. Journal.*

**Ice to the Abdomen in Typhoid Fever.**—At a recent *seance* of *La Societe Medicale des Hopitaux*, M. Labbe called attention to the efficacy of ice application to the abdomen in typhoid fever, complicated or not. He related the case of a young girl attaked with typhoid, whose temperature exceeded 104°, and who appeared at the last extremity, who under the influence of this treatment was perfectly cured. M. Labbe claims for this procedure a considerable lowering of the temperature and a notable amelioration of all the other symptoms.

**Cough Mixture.**—

| | | |
|---|---|---|
| ℞ | Syr. pruni virg. | ℥ ij |
| | Syr. scillæ | ℥ j |
| | Syr. ipecac | ℥ j |
| | Tinct. opii camphorat | ℥ j |
| | Carb. ammoniæ | ʒ ijss |
| | Aq. qs. ad. | ℥ vi |

Dose, a teaspoonful every two or three hours.—*Ex.*

**Chloroform Cough Mixture.**—

| | | |
|---|---|---|
| ℞ | Morphia acet | gr. iij |
| | Tinct. belladonnæ | ʒ ij |
| | Spts. chloroformi | ʒ vi |
| | Syr. senegæ | ℥ j |
| | Syr. pruni virg. ad | ℥ iv |

Dose, one teaspoonful three times per day.

—*Arkansas Med. Monthly.*

**Treatment of Poisoning by Rhus Toxicodendron.**—Dr. J. H. Egan, of Tennessee, writes :

My treatment for this distressing eruption has been, for years past, the topical application of fluid extract grindelia robusta, either pure or diluted as the exigencies of the case demanded. In no case have I failed to afford immediate relief.

**Eucalyptus for Wounds.**—The fluid extract of eucalyptus is strongly recommended as a local application for wounds. Having antiseptic properties it is likely to come into general use as an application to gangrenous surfaces and offensive ulcerations of all kinds.

**Picrotoxin in Night Sweats.**—In doses of 1–120 to 1–60 of a grain, Dr. William Murrell found picrotoxin to arrest the night sweats of phthisis, in nineteen out of twenty cases. He gave the dose mentioned in simple aqueous solution, at bedtime.—*Practitioner.*

*Dr. C. M. Gibson writes*—Will some of my medical friends, through the "Record", give their experience with the use of Pessaries for prolapsed uteri? Also, any other modes of treatment of the same, &c. Please mention what kind of Pessary was used, and oblige a subscriber ot the "Southern Medical Record."

